# Prognostic cellular senescence-related lncRNAs patterns to predict clinical outcome and immune response in colon cancer

**DOI:** 10.3389/fimmu.2024.1450135

**Published:** 2024-09-02

**Authors:** Lichao Cao, Fang Chen, Long Xu, Jian Zeng, Yun Wang, Shenrui Zhang, Ying Ba, Hezi Zhang

**Affiliations:** ^1^ Shenzhen Nucleus Gene Technology Co., Ltd., Shenzhen, Guangdong, China; ^2^ Shenzhen Nucleus Huaxi Medical Laboratory, Shenzhen, Guangdong, China; ^3^ Shanghai Nucleus Biotechnology Co., Ltd., Shanghai, China; ^4^ Department of Gastroenterology and Hepatology, Shenzhen University General Hospital, Shenzhen, Guangdong, China; ^5^ Marshall Laboratory of Biomedical Engineering, Shenzhen University, Shenzhen, Guangdong, China; ^6^ Longhua Innovation Institute for Biotechnology, College of Life Sciences and Oceanography, Shenzhen University, Shenzhen, Guangdong, China

**Keywords:** colon cancer, cellular senescence, lncRNAs, MYOSLID, immune response

## Abstract

**Background:**

Cellular senescence (CS) is believed to be a major factor in the evolution of cancer. However, CS-related lncRNAs (CSRLs) involved in colon cancer regulation are not fully understood. Our goal was to create a novel CSRLs prognostic model for predicting prognosis and immunotherapy and exploring its potential molecular function in colon cancer.

**Methods:**

The mRNA sequencing data and relevant clinical information of GDC TCGA Colon Cancer (TCGA-COAD) were obtained from UCSC Xena platform, and CS-associated genes was acquired from the CellAge website. Pearson correlation analysis was used to identify CSRLs. Then we used Kaplan–Meier survival curve analysis and univariate Cox analysis to acquire prognostic CSRL. Next, we created a CSRLs prognostic model using LASSO and multivariate Cox analysis, and evaluated its prognostic power by Kaplan–Meier and ROC curve analysis. Besides, we explored the difference in tumor microenvironment, somatic mutation, immunotherapy, and drug sensitivity between high-risk and low-risk groups. Finally, we verified the functions of MYOSLID in cell experiments.

**Results:**

Three CSRLs (AC025165.1, LINC02257 and MYOSLID) were identified as prognostic CSRLs. The prognostic model exhibited a powerful predictive ability for overall survival and clinicopathological features in colon cancer. Moreover, there was a significant difference in the proportion of immune cells and the expression of immunosuppressive point biomarkers between the different groups. The high-risk group benefited from the chemotherapy drugs, such as Teniposide and Mitoxantrone. Finally, cell proliferation and CS were suppressed after MYOSLID knockdown.

**Conclusion:**

CSRLs are promising biomarkers to forecast survival and therapeutic responses in colon cancer patients. Furthermore, MYOSLID, one of 3-CSRLs in the prognostic model, could dramatically regulate the proliferation and CS of colon cancer.

## 1 Introduction

Colon cancer, one of the most-diagnosed cancer, is the second most common causes of cancer-related death globally (1). According to the latest cancer statistics from the American Cancer Society, there were 81,860 colon cancer cases in males and 71,160 cases in females, with 52,550 deaths in 2023 (2). Recently, despite the rapid development of cancer screening methods (3), the incidence of colon cancer remains high, and effective therapeutic targets are still few. In addition, the AJCC TNM staging system, as a prognostic signature for colon cancer patients, is constantly updated, but there are still differences in prognosis among patients with the same clinicopathologic characteristics (4, 5). Therefore, further exploration of specific and sensitive prognostic biomarkers and possible therapeutic targets is essential to ameliorate the clinical outcome and treatment of colon cancer.

Cellular senescence (CS) defined as a state of permanent cell cycle termination (6, 7). Currently, there are 8 types of CS phenotypes, which are mainly triggered by DNA damage response, involvement of cycle-related kinase inhibitors, enhanced secretion of pro-inflammatory factors and tissue repair factors, induction of anti-apoptotic genes, metabolic changes, and endoplasmic reticulum stress (8, 9). Recently, there has been increasing evidence that CS not only has a suppressor effect on tumor (10), but that senescent cells can also accelerate tumor growth by promoting immune escape (11, 12). In the third edition of cancer hallmarks proposed in 2022, senescent cells are recognized as one of novel cancer hallmarks (13). However, few reports have explored the role of CS in the occurrence, development and treatment of colon cancer (14). Therefore, further screening of CS-associated genes based on clinical samples is necessary for the diagnosis and prognosis of colon cancer.

Long noncoding RNA (lncRNA) with more than 200 nucleotides in length, do not have the ability to encode proteins (15). LncRNA has been revealed to play a key role in regulating the physiological activity of cancer cells (16, 17). Furthermore, lncRNA is an ideal tumor biomarker with high specificity and sensitivity that are easy to repeat detection (18). LncRNA plays a functional role in development of CS. Activation of p53 is a key initiating event in CS (19). Several lncRNAs has been reported as regulators or mediators of the p53 pathway, such as lncRNA-H19 and lncRNA DANCR (20, 21). Besides, lncRNA UCA1, as a pro-senescence agent, has been established as an oncogene in several malignancies (22). More importantly, CS-related lncRNAs (CSRLs) were regarded as potential biomarkers for assessing the prognosis of multiple cancers, such as hepatocellular carcinoma (23), lung adenocarcinoma (24), breast cancer (25). Moreover, some study demonstrated that lncRNA PURPL suppressed basal p53 levels, promoting tumorigenicity of colorectal cancer cells, thereby contributing to the pro-survival phenotype of senescent cells (26). However, there are currently few studies about CSRLs in colon cancer (27). Given this, the identification of prognostic CSRLs is important for the prognosis and treatment of colon cancer.

Here, we aimed to explore the prognostic significance of CSRLs in colon cancer. Specifically, a CSRLs prognostic model was constructed to evaluate the performance in the diagnosis, prognosis and therapeutic response for colon cancer.

## 2 Methods

### 2.1 Data acquiring and preparation

The RNA sequencing data of GDC TCGA Colon Cancer (TCGA-COAD) cohort (including 469 tumor tissues and 41 normal tissues) and corresponding clinical information, gene expression profiles and mutation profiling data were downloaded from the UCSC Xena platform (https://xenabrowser.net/datapages/). Then, we used the GENCODE website to identified 15,088 lncRNAs via the lncRNA annotation file. Subsequently, transcriptome profiles were used to extract expression matrixes for lncRNAs. In addition, CellAge (https://genomics.senescence.info/download.html#cellage) provided a list of 601 CS-related genes.

### 2.2 Identification of CSRLs

Differentially expressed genes (DEGs) between normal and cancer tissues were screened out according to |log_2_ fold change (FC)| > 0.585 and adjusted *P*-value < 0.05. Then, venn diagram was used to show overlapping CS-related DEGs between DEGs and CS-related genes. Pearson correlation analysis was performed based on CS-related DEGs and lncRNAs expression levels to identify CSRLs with |Pearson correlation coefficient| > 0.5 and *P*-value of < 0.001 (28, 29).

### 2.3 Creation and validation of CSRLs prognostic model

Transcriptome expression data of 469 tumor samples in TCGA-COAD cohort were obtained, among which 37 samples without survival or phenotypic information were excluded. Remaining samples (n=432) considered as the entire cohort. The information of the entire cohort is showed in **Supplementary Table 1**. Then, the entire cohort was randomly classified into training (n=216) or test (n=216) sets at a 1:1 ratio. Next, a prognostic risk model was generated in the training cohort and validated in test and entire cohorts, respectively. First, the prognostic CSRLs were obtained by the association between the CSRLs expression level and patients’ overall survival (OS) using Kaplan–Meier analysis (*p* < 0.05). Subsequently, univariate Cox regression with a *P*-value of < 0.05 was applied to further filtrate optimal prognostic CSRLs among the above filtered candidate prognostic CSRLs. The least absolute shrinkage and selection operator (LASSO) analysis was applied to the above prognostic CSRLs to avoid over-fitting. Then a CSRLs prognostic model was established by applying multivariate Cox regression analysis. The formula for the CSRLs prognostic model was built to forecast patient survival (28):

$\text{risk core}=\sum \;Cox\;coefficient\;of\;gene\;x^{i}\;*\;expression\;value\;ofgene\;x^{i}.$

The regression coefficient was obtained from the multivariate Cox results.

The receiver operating characteristic (ROC) curve was applied to estimate the predictive accuracy of the prognostic model via the *survivalROC* R package, which was reflected by quantifying area-under-curve (AUC) for assessing the CSRLs prognostic model’s sensitivity as well as specificity. Meantime, the optimum critical point of the ROC curve is regarded as the best cutoff value. Colon cancer patients were divided into the high-risk group and the low-risk group based on the cutoff value. Kaplan-Meier curves were plotted using the *survminer* R package to show the relationship between high-risk and low-risk groups and prognosis.

Besides, the test and entire cohorts were performed to assess the model feasibility, respectively. The verification measure was the same as above.

Additionally, the relationship between the CSRLs prognostic model and the pathological stages, microsatellite status, and TNM stages were examined by the Wilcoxon test and Kruskal-Wallis test.

### 2.4 Function analysis of the 3 prognostic CSRLs

EnrichR is a Gene Set Enrichment method that speculates biological information by enriching input gene sets that represent biological functions or functional pathways (30). We used the ‘enrichR’ package to perform Gene Ontology (GO) and Kyoto Encyclopedia of Genes and Genomes Enrichment (KEGG) enrichment analyses of the 3 lncRNAs-correlated CS-associated DEGs in R.

### 2.5 Relationship between immune cell infiltration and the model

Investigation the immune cell infiltration can provide prognostic value and guide immunotherapy in colon cancer (31). The CIBERSORT algorithm was performed to obtain the proportions of 22 types of tumor-infiltrating immune cells (32). The unpaired *t*-test was applied to compare the proportions of tumor-infiltrating immune cells between the high- and low-risk groups. Kaplan–Meier curve was performed to assess the correlation between OS and significant differential immune cell types (*P*-value < 0.01).

### 2.6 Genetic alterations analysis

Mutation data from colon cancer patients were obtained from TCGA and the R package “maftools” was used to visualize the gene mutation landscape in different subgroups.

### 2.7 Exploring immunotherapy response

Wilcoxon test was applied to compare the mRNA levels of CD274, PDCD1, CTLA4, HAVCR2, LAG3, and TIGIT between the high- and low-risk groups. Then, we calculated the tumor mutation burden (TMB) value of different subgroups using the R package “maftools” and performed immunotherapy analysis. Finally, the *oncoPredict* R package was applied to compare the IC50 values of 8 chemotherapeutic drugs between different risk groups.

### 2.8 Cell line culture and transfection

American Type Culture Collection (ATCC) provided HCT116 and SW480 cells. These cells were grown in McCoy’s 5A or Leibovitz’s L-15 medium (Gibco, United States) with 10% fetal bovine serum (Gibco, United States). All cells were cultured in a 37°C and 5% CO_2_ cell incubator. Follow manufacturer’s instructions, jetPRIME^®^ (Polyplus, France) was performed to transfect cells with ASOs (Tsingke Biotech, Beijing, China). Sequences of ASOs were listed in **Supplementary Table 2**.

### 2.9 Quantitative real-time polymerase chain reaction

TRIzol reagent was applied to extract total RNA from cell lines. Then, the obtained RNAs were used for cDNA synthesis using the Hifair^®^ III 1st Strand cDNA Synthesis SuperMix (YESEN, Shanghai, China). Gene expression was quantified by conducted with Hieff^®^ qPCR SYBR Green Master Mix (YESEN, Shanghai, China). The relative quantitative value were calculated with the 2^−ΔΔCt^ method. The primer sequences were shown in **Supplementary Table 3**.

### 2.10 Cell counting kit-8 assay

The cells were seeded into a 96-well plate at a density of 1000 cells/well for 24 h. Then, 10 μL CCK-8 reagent (Yeasen, Shanghai, China) was added to each well at the indicated time (24h, 48h, 72h, 96h) and incubated for 10 min. Absorbance was measured at 450nm.

### 2.11 Statistical analysis

GraphPad Prism 8.0 software was used to perform statistical analysis. Student’s t test was applied to assess the differences between the two groups. Data were expressed as the mean ± standard deviations (SD). *P* < 0.05 was set as the significance level.

## 3 Results

### 3.1 Screening CSRLs in colon cancer

**Figure 1:**
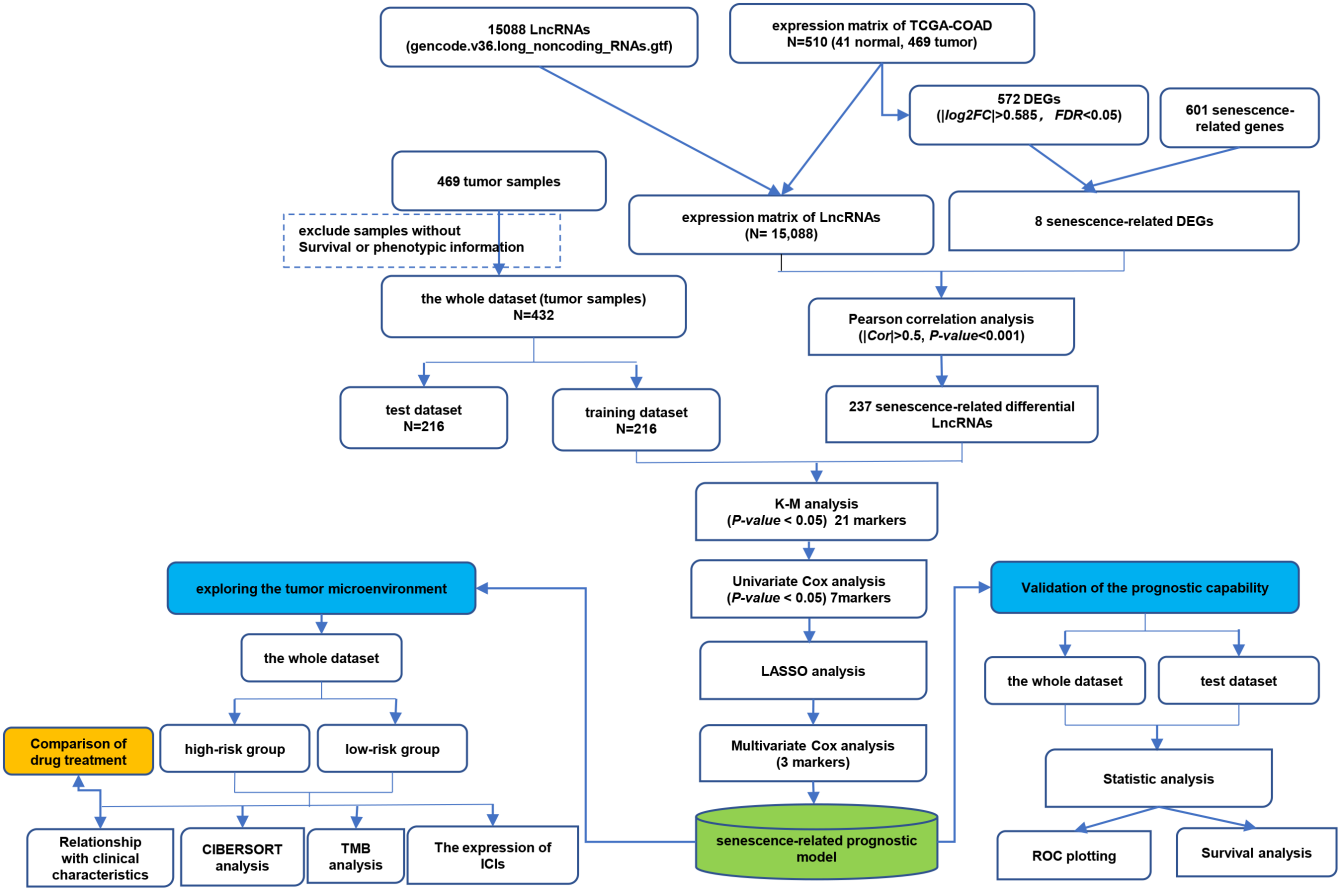
The technical flow chart of this study.

The detailed flowchart is shown in **Figure 1**. In this study, we acquired transcriptome data of 510 colon cancer samples (including 41 normal and 469 tumor samples) from the TCGA-COAD cohort. There were 572 DEGs (276 up-regulated and 296 down-regulated) between normal and tumor tissues (**Supplementary Table 4**). Then, we obtained 601 CS-related genes from CellAge database. The 8 overlapping genes were considered CS-related DEGs (**Supplementary Figure S1**). Subsequently, we performed a Pearson correlation analysis of the obtained 8 CS-related DEGs and 15,088 lncRNAs to obtain CSRLs (**Supplementary Table 5**). Finally, 237 CSRLs were identified.

### 3.2 Construction and validation of the CSRLs prognostic model

**Figure 2:**
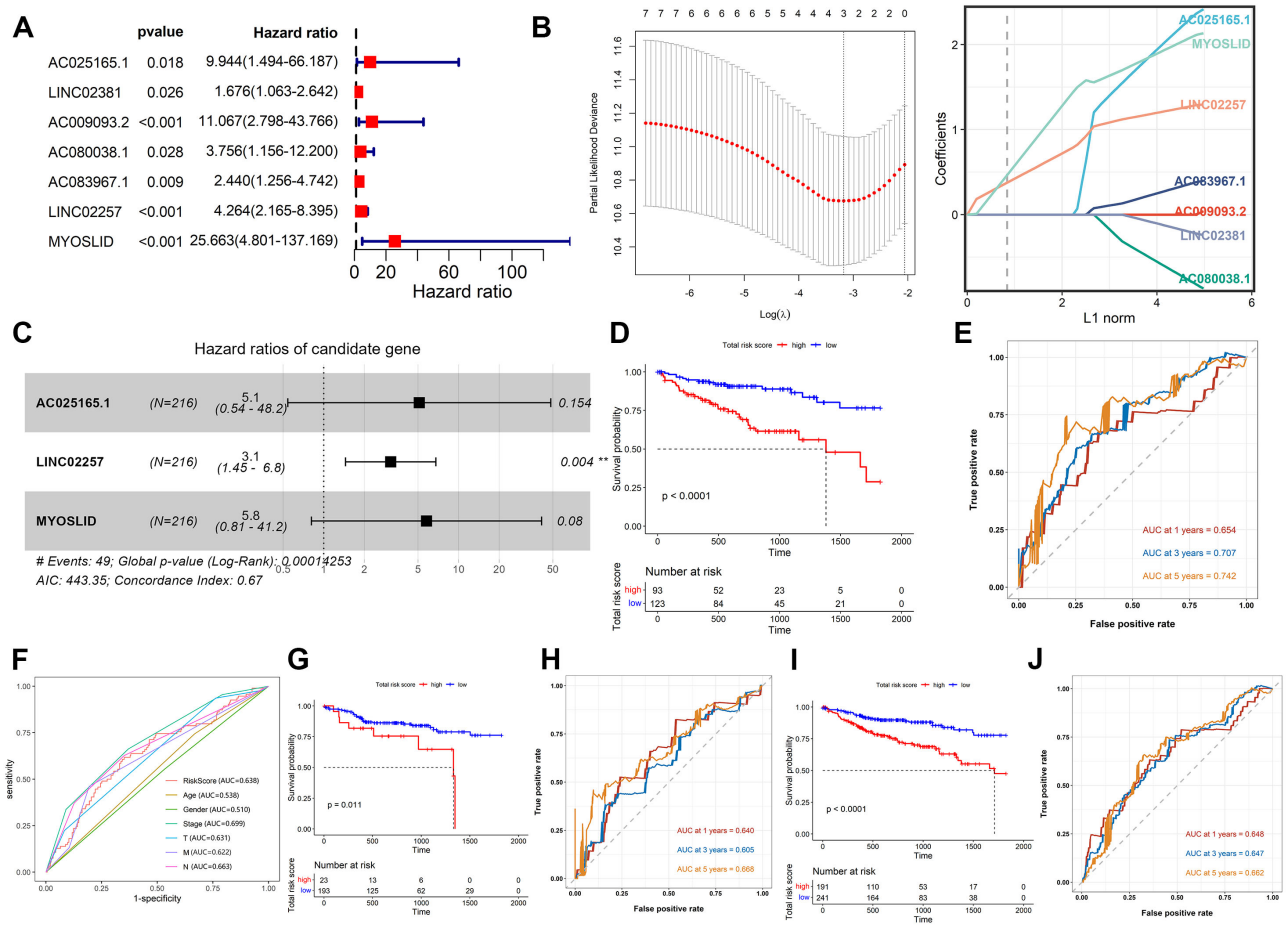
Construction and validation of the CSRLs prognostic model. **(A)** Univariate Cox regression analysis. **(B)** LASSO regression analysis. **(C)** Multivariate Cox regression analysis. Kaplan-Meier survival analyses of the CSRLs prognostic model in the **(D)** training cohort, **(G)** test cohort and **(I)** entire cohort. ROC curves indicated the potential of the CSRLs prognostic model in predicting 1-, 3- and 5-year OS in the **(E)** training cohort, **(H)** test cohort and **(J)** entire cohort. **(F)** ROC curves comparing the prognostic accuracy of the risk score and other clinical characteristics in the training cohort.

Using the Kaplan–Meier analysis, the expression levels of 21 CSRLs were significantly associated with patient’s OS (*P* < 0.05; **Supplementary Figure S2**) in the training cohort. Then, univariate Cox regression analysis showed 7 CSRLs are associated with prognosis in the training cohort (*P* < 0.05; **Figure 2A**). LASSO regression analysis has confirmed 7 CSRLs have the maximum prognostic value (**Figure 2B**). Subsequently, the multivariate Cox regression analysis was applied to establish a senescence-related prognostic model composed of 3 CSRLs (AC025165.1, LINC02257 and MYOSLID) based on the training cohort (**Figure 2C**). Colon cancer patients were classified into high- and low-risk groups according to the cutoff value of ROC curves. **Figure 2D** showed patients with high-risk group has shorter survival times than those in low-risk group in the training cohort (*P* < 0.0001). Moreover, the AUC values at 1-, 3- and 5-year were 0.654, 0.707 and 0.742 in the training cohort, respectively (**Figure 2E**), demonstrating the predictive reliability of the CSRLs prognostic model. We also constructed an ROC curve to validate the prognostic accuracy of this prognostic model compared to other clinical characteristics (**Figure 2F**). Furthermore, we also validated the prognostic power of the model in the test cohort and the entire cohort (**Figures 2G–J**). The AUC values at 0.64, 0.605, and 0.668 for 1-, 3- and 5-year in the test cohort, accordingly (**Figure 2H**); the AUC values at 0.648, 0.647, and 0.662 for 1-, 3- and 5-year in the entire cohort, respectively (**Figure 2J**). These results indicated that the CSRLs prognostic model can predict the prognosis of colon cancer.

### 3.3 Relationship between the CSRLs prognostic model and the clinicopathological characteristics

**Figure 3:**
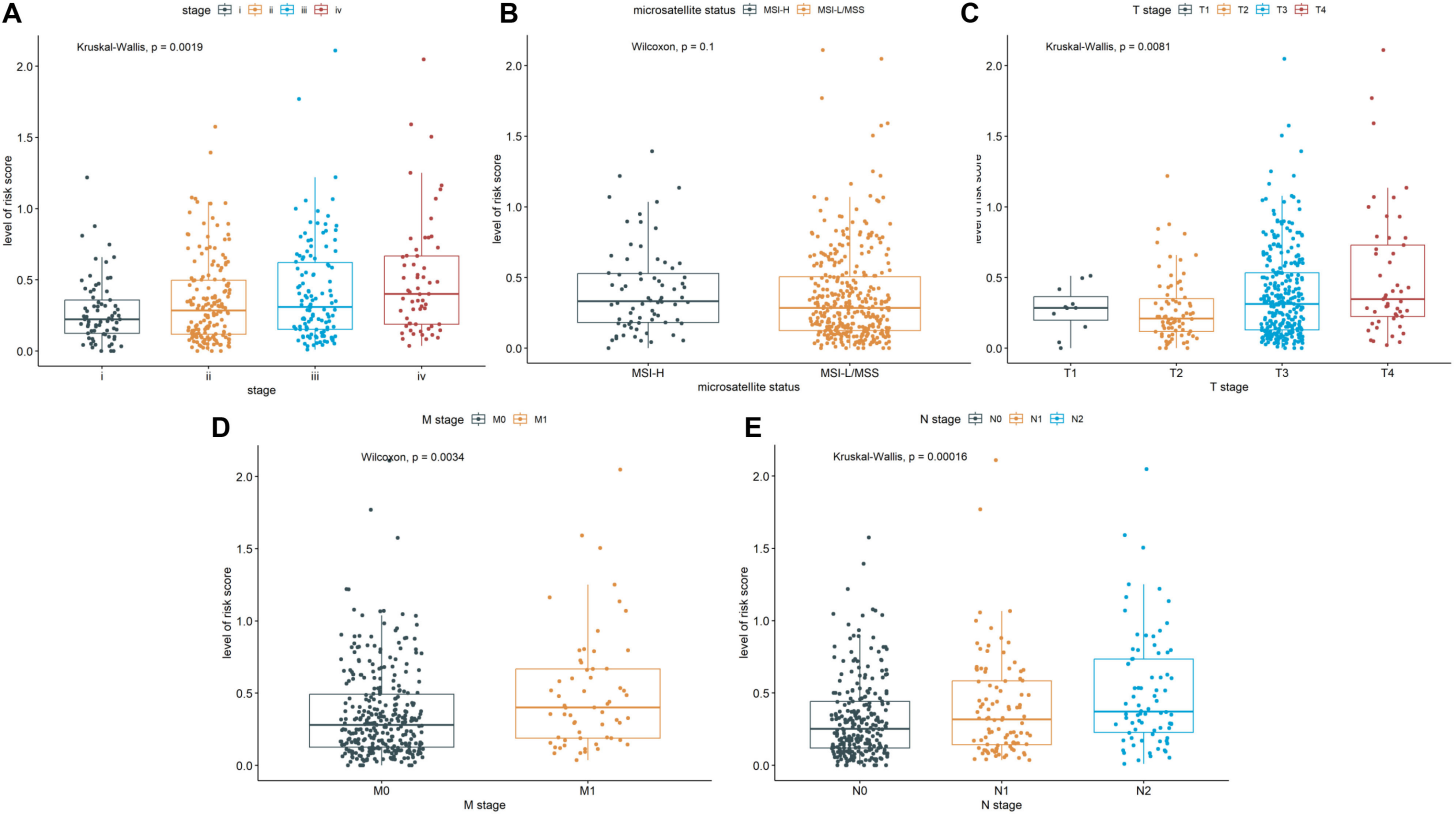
Correlation of the CSRLs prognostic model and the clinicopathological characteristics, such as **(A)** pathological stage, **(B)** microsatellite status, and **(C–E)** TNM stages based on the entire cohort.

We further explored whether there were differences in risk scores for different clinical features. There was differences in risk scores among pathological stages (Stage I, Stage II, Stage III, Stage IV), T stages (T1, T2, T3, T4), M stages (M0, M1), and N stages (N0, N1, N2) (**Figures 3A, C–E**). In general, patients with advanced stage tumors also had higher risk scores. In contrast, the risk scores exhibited no differences between MSI-H and MSI-L (**Figure 3B**). These findings demonstrated that the CSRLs prognostic model has outstanding potential to predict clinical characteristics in patients with colon cancer.

### 3.4 Function analysis of the 3 prognostic CSRLs

**Figure 4:**
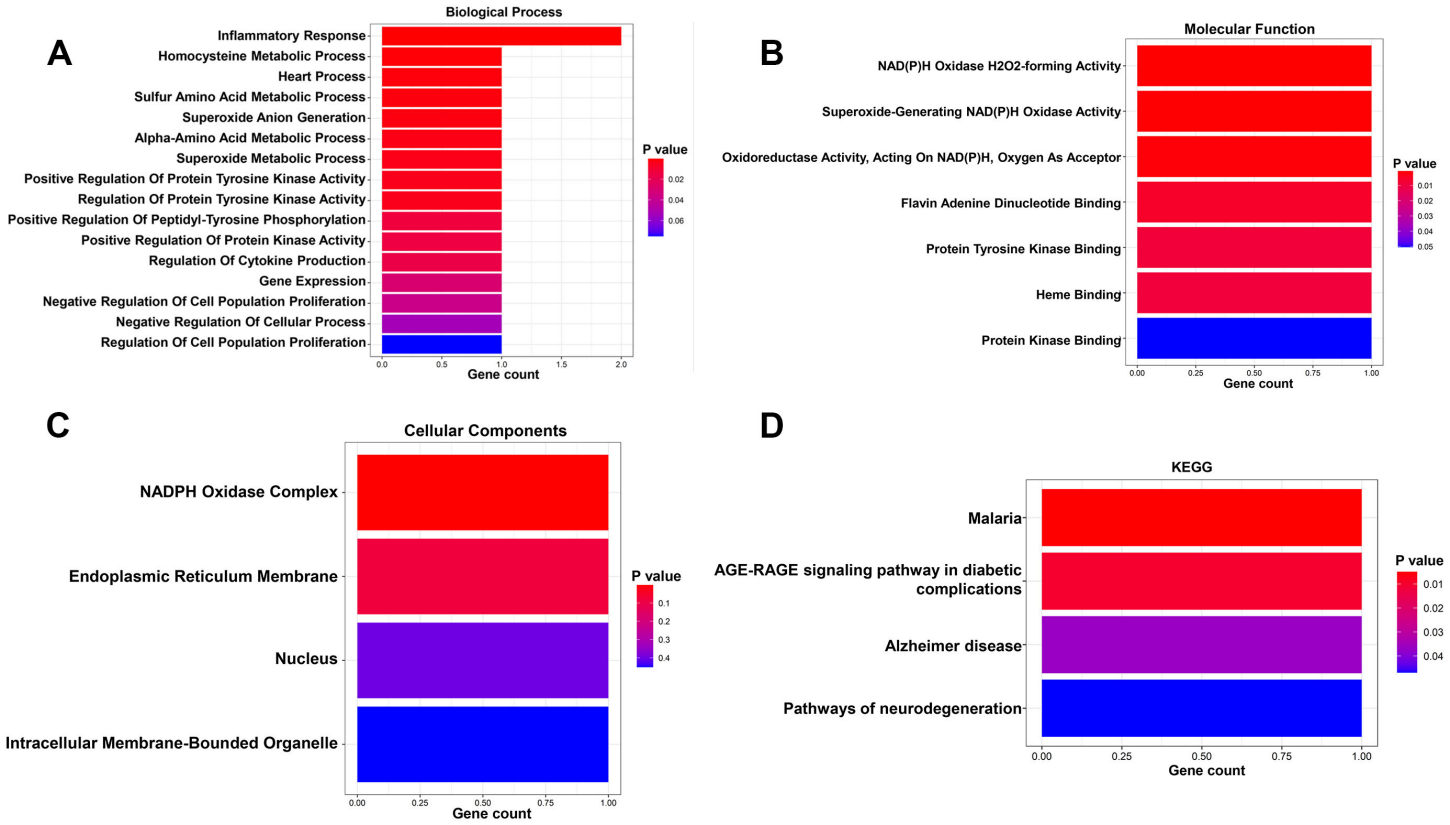
GO and KGEE analysis of the 3 prognostic CSRLs. **(A)** Biological process of the 3 CSRLs-associated DEGs. **(B)** Molecular function of the 3 CSRLs-associated DEGs. **(C)** Cellular components of the 3 CSRLs-associated DEGs. **(D)** KEGG of the 3 CSRLs-associated DEGs.

Our results showed that AC025165.1, LINC02257 and MYOSLID may be involved in the regulation of 2 CS-related DEGs (ACKR1 and NOX4). The 2 CS-related DEGs were significantly enriched in the biological process terms inflammatory response and homocysteine metabolic process (**Figure 4A**). The 2 CS-related DEGs were significantly enriched in the molecular function terms NAD(P)H oxidase H2O2-forming activity and superoxide-generating NAD(P)H oxidase activity (**Figure 4B**). The 2 CS-related DEGs found to be involved in the Cellular Component: NADPH oxidase complex and endoplasmic reticulum membrane (**Figure 4C**). KEGG pathway showed 2 CS-related DEGs were enriched in AGE-RAGE signaling pathway in diabetic complications (**Figure 4D**).

### 3.5 Correlation of the CSRLs prognostic model with immune characteristics

**Figure 5:**
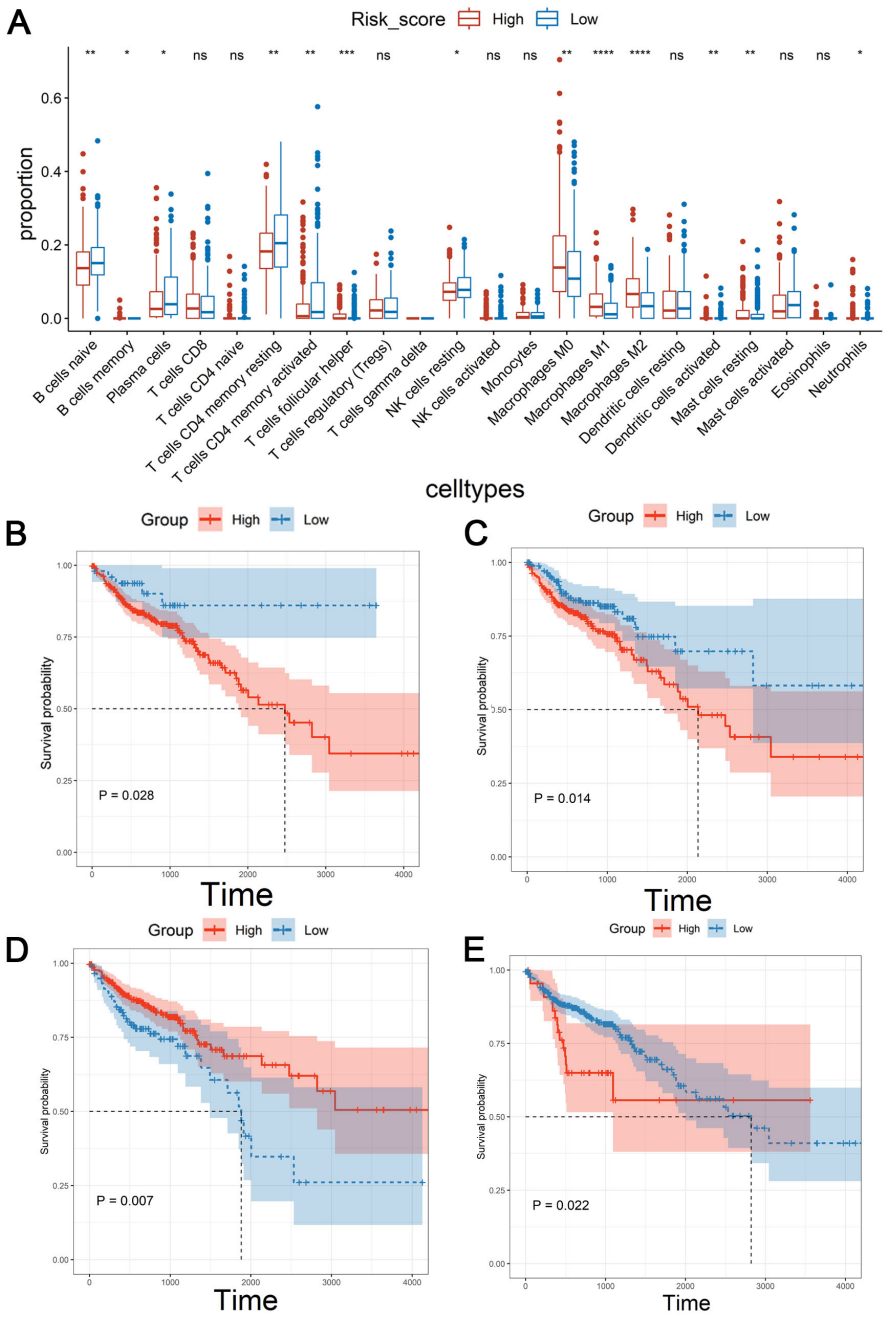
Correlation of the CSRLs prognostic model with immune microenvironment based on the entire cohort. **(A)** Immune cell infiltration analysis. * indicated *P*<0.05, ** indicated *P*<0.01, *** indicated *P*<0.001, **** indicated *P*<0.0001, ns indicated no significant difference. Kaplan-Meier survival analyses of the relationship between the level of **(B)** M0 macrophages, **(C)** resting NK cells, **(D)** M1 Macrophages, and **(E)** naïve B cells with patients’ OS.

Immune microenvironment is a key factor affecting tumor growth and patient prognosis (33). As shown in **Figure 5A**, there were the significant differences in naïve B cells, memory B cells, plasma cells, CD4 memory-resting T cells, CD4 memory-activated T cells, follicular helper T cell, resting NK cells, M0 macrophages, M2 macrophages, M3 macrophages, activated dendritic cell, resting mast cells and neutrophils between the low-risk group and high-risk group. Among them, the poor prognosis of patients was associated with the high level of M0 macrophages or resting NK cells (**Figures 5B, C**). Conversely, down-regulated M1 Macrophages or naïve B cells were associated with a poor prognosis (**Figures 5D, E**). These results suggested that the CSRLs prognostic model might reflect the immune microenvironment status in patients with colon cancer.

### 3.6 Cancer type-specific genomic variations in the CSRLs prognostic model

**Figure 6:**
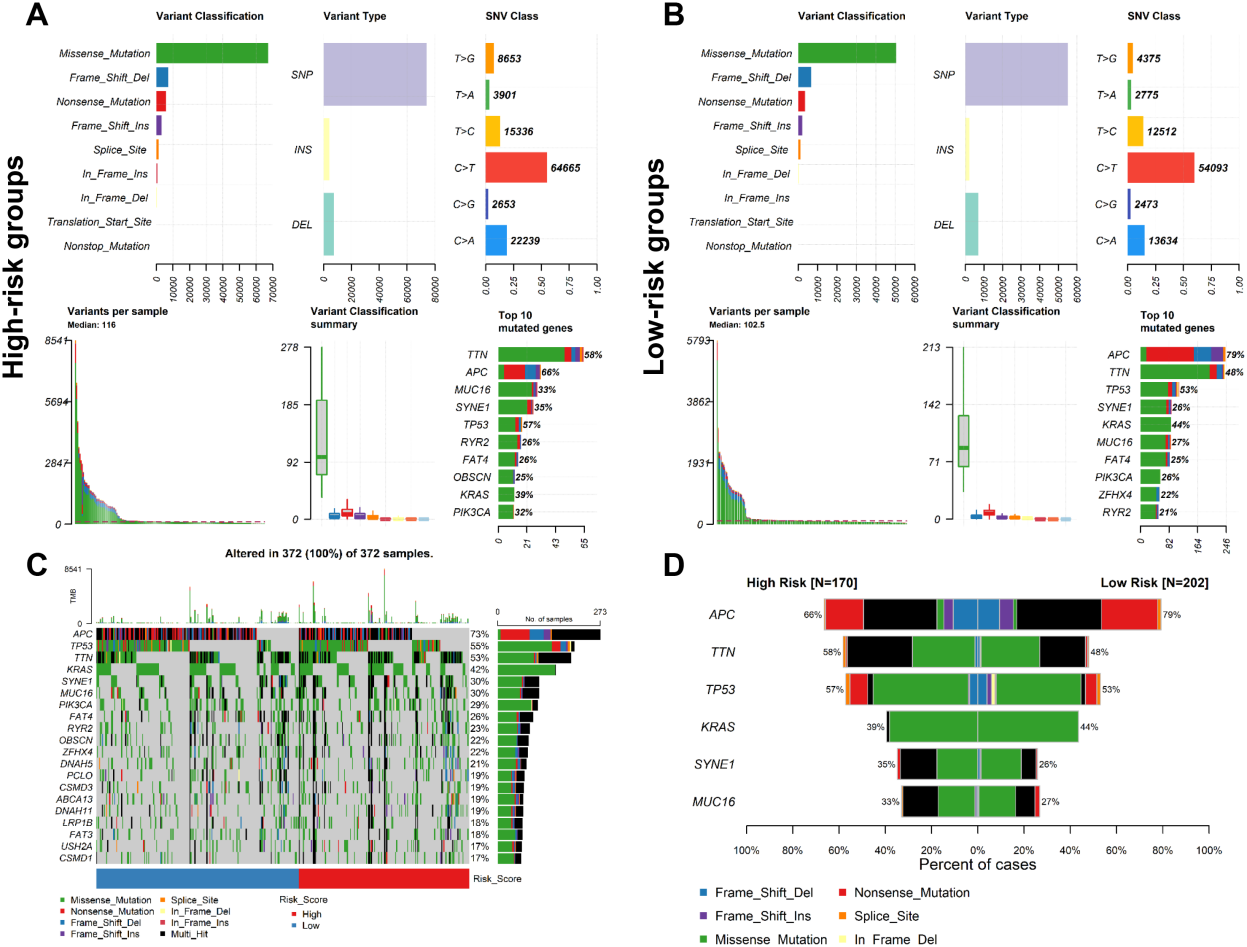
Analysis of mutation profiles in low- and high-risk groups based on the entire cohort. Mutation characteristics of **(A)** high- and **(B)** low-risk group. **(C)** The mutation profiles of all patients. **(D)** Comparison of the mutation rate between two risk groups.

To investigate the gene mutation for the CSRLs prognostic model in colon cancer, we used *maftools* R package to explore the mutation profiles of the low-risk and high-risk groups. While the top 10 mutated genes in high-risk and low-risk groups were similar, their ranking differed. Additionally, the median number of mutations in high-risk group was higher than in low-risk group (116 vs. 102.5). In high-risk and low-risk groups, the most common variant classification was missense mutation, the most common variant type was single nucleotide polymorphisms (SNP), and the most common single nucleotide variants (SNVs) class was C>T (**Figures 6A, B**). Moreover, we also examined the top 20 significantly mutated genes in all patients (**Figure 6C**). Generally, APC, had a relatively higher mutation rate in the low-risk groups (79% vs. 66%), while TTN presented a relatively higher mutation rate in the high-risk group (58% vs. 48%) (**Figure 6D**). These genomic alterations may be associated with differences in senescence cells between low-risk and high-risk patients.

### 3.7 The role of CSRLs prognostic model in clinical treatment

**Figure 7:**
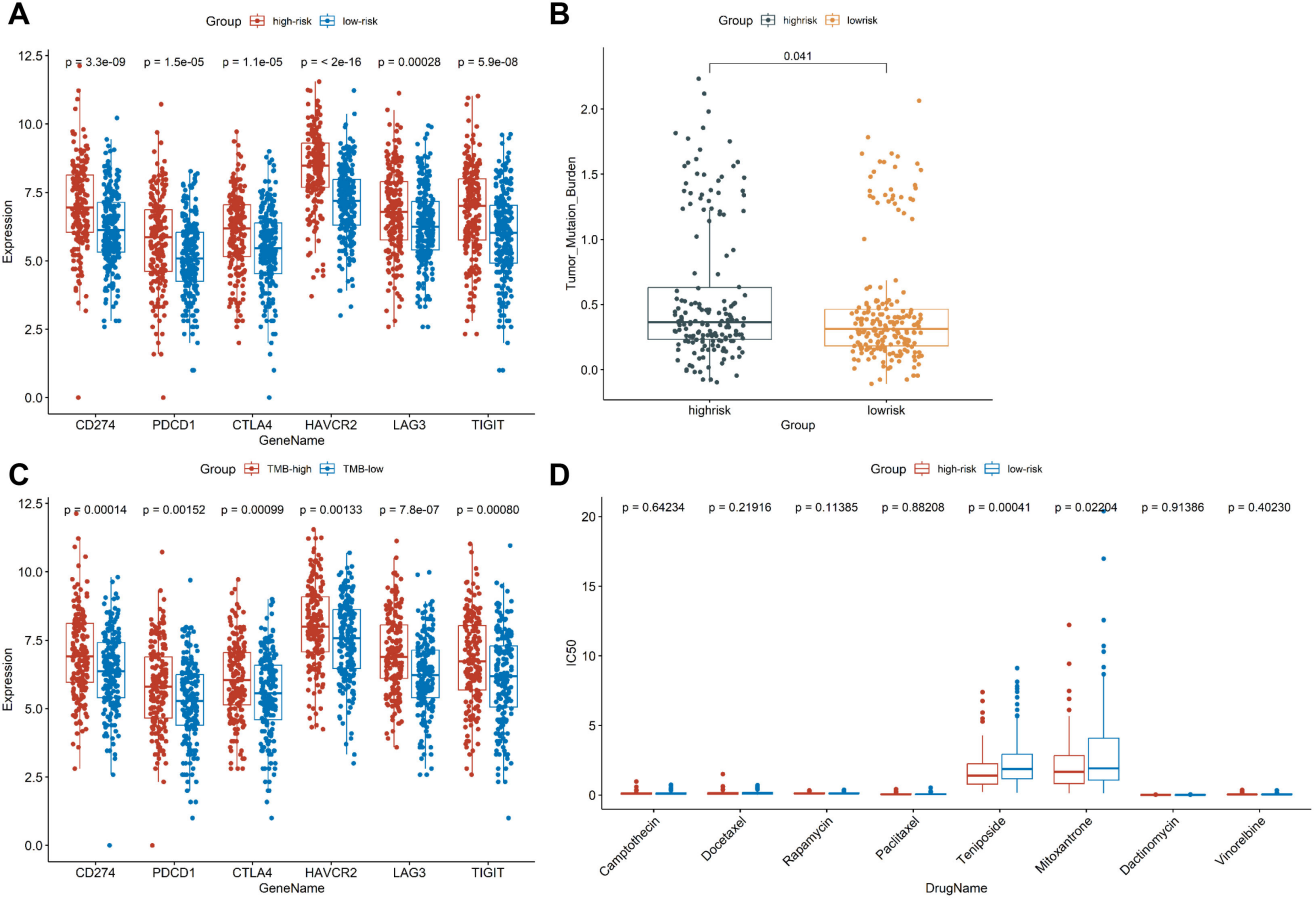
Exploring the role of CSRLs prognostic model in clinical treatment. **(A)** Comparison of the expression of immunosuppressive point biomarkers between low- and high-risk groups. **(B)** Comparison of TMB values between low- and high-risk groups. **(C)** Comparison of the expression of immunosuppressive point biomarkers between TMB-low and TMB-high groups. **(D)** Comparison of the IC50 values of chemotherapy drugs between low- and high-risk groups.

Since immune checkpoint inhibitor (ICI) has been shown to have beneficial effects in the treatment of colon cancer in clinical trials, we further investigated the role of immunosuppressive point biomarkers in the model. The results showed that the expression of immunosuppressive point biomarkers in the high-risk group was higher than that those with a low-risk group (**Figure 7A**), suggesting that patients in high-risk group may be better candidates for immunosuppressive therapy. Additionally, TMB has been proved to be an important indicator for predicting the clinical benefits of immunotherapy. There was a significant difference in TMB between the high-risk and low-risk groups (**Figure 7B**). Similarly, the expression of immunosuppressive point biomarkers in the TMB-high group was higher than that those with a TMB-low group (**Figure 7C**). In addition to ICI treatment, chemotherapy is also a common treatment for colon cancer. The results demonstrated that the high-risk group marked clinical benefits from Teniposide (*P*=0.00041) and Mitoxantrone (*P* = 0.02204) compared to low-risk group, but no significant difference with other 6 chemotherapeutic drugs between high-risk and low-risk groups (**Figure 7D**). Collectively, our prognostic model is suitable for providing immunotherapeutic strategies and predicting drug sensitivity for colon cancer patients.

### 3.8 Knockdown of MYSOLID inhibited the cell proliferation and CS of colon cancer

**Figure 8:**
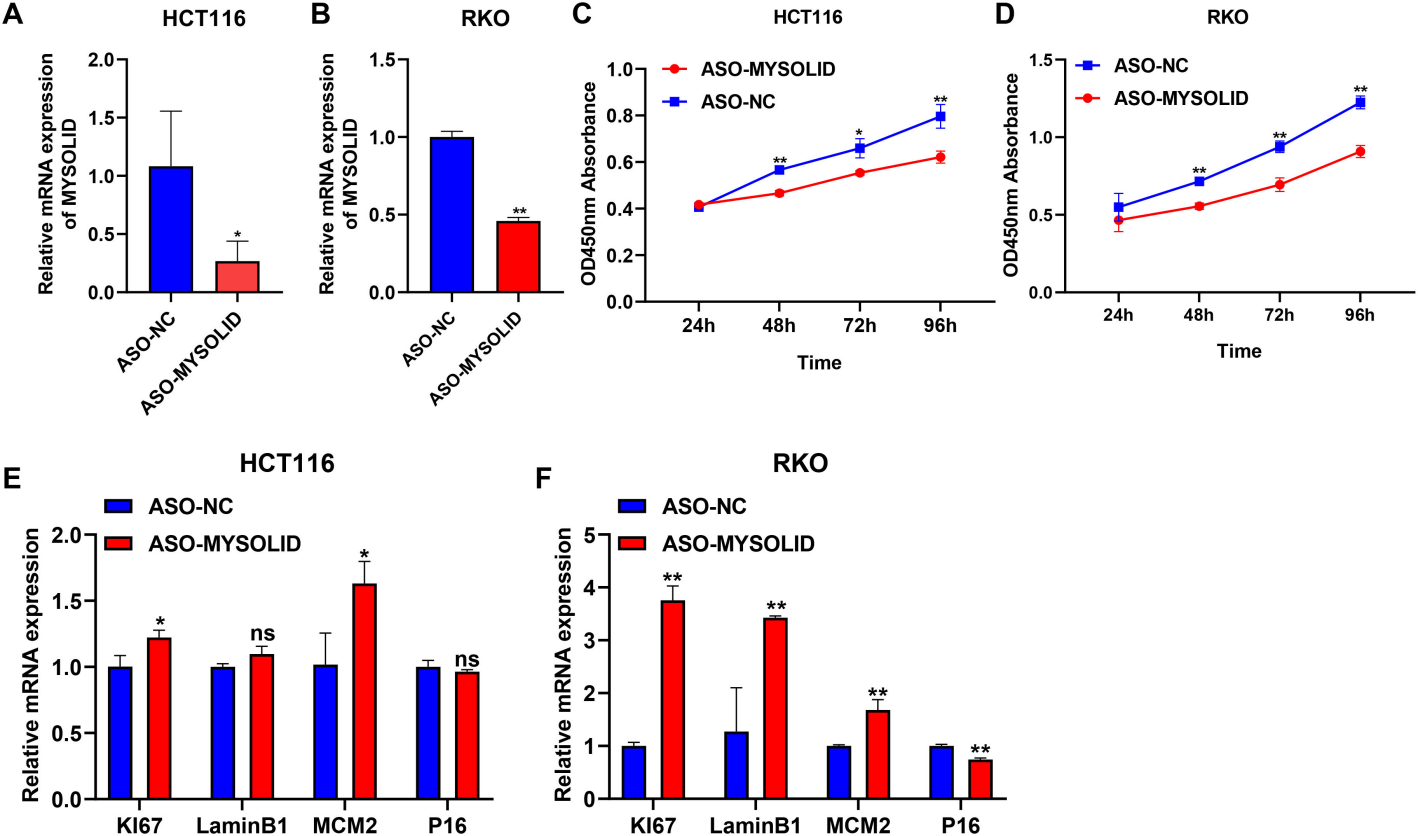
Exploring the role of MYSOLID in colon cancer. q-PCR verified the efficiency of MYSOLID knockdown in **(A)** HCT116 and **(B)** RKO cells. The activity of cells was markedly down-regulated following MYSOLID knockdown in **(C)** HCT116 and **(D)** RKO cells. q-PCR verified the expression of CS-related biomarkers following MYSOLID knockdown in **(E)** HCT116 and **(F)** RKO cells. * indicated *P*<0.05, ** indicated *P*<0.01, ns indicated no significant difference.

CS has been proved to depress the development of colon cancer cells (34) and can also enhance the progression of colon cancer (35), which may be correlated with the fact that it is highly heterogeneous (36). Here, we selected MYOSLID to examine the relationship between CS and colon cancer. MYOSLID has been revealed to be highly expressed in colon cancer cell lines (RKO and HCT116) and accelerate the malignant activity of colon cancer cells (37, 38). Therefore, we synthesized 3 pairs of ASO sequences and mixed them to interfere with MYOSLID expression. In both the HCT116 and RKO cell lines, MYOSLID dramatically reduced (**Figures 8A, B**). The activity of colon cancer cells was observably decreased following MYOSLID knockdown in HCT116 and RKO cell lines (**Figures 8C, D**), which is similarly to other reports (37). Subsequently, we further verified the effect of MYOSLID knockout on biomarkers of CS. The results demonstrated the expressions of KI67 and MCM2 were considerably increased following MYOSLID knockdown in HCT116 (**Figure 8E**). Similarly, MYOSLID knockdown dramatically up-regulated the expressions of KI67, LaminB1, and MCM2, and significantly reduced the expression of P16 in RKO cell lines (**Figure 8F**). Taken together, MYOSLID promoted cell proliferation and CS of colon cancer cells.

## 4 Discussion

Colon cancer is a recognized malignant tumor with a very high mortality rate. The occurrence of colon cancer is mainly associated with two types of precursor polyps produced by two distinct pathways (39). However, the progression of colon cancer is a multistep process involving changes in many endogenous and exogenous factors, such as tumor microenvironment (TME) (40), immune escape (41), alteration of intestinal flora (42), and environmental factors (43). Accumulating evidence indicated that CS is a key process in cancer progression and treatment (44). However, studies of CS and colon cancer are rare. Nowadays, lncRNA, as a stable expression biomarker with high detection sensitivity, have been used in the early diagnosis of a variety of cancers (45, 46). Therefore, we successfully established a CSRLs prognostic model and provided reliable early prognostic indicators for colon cancer.

Here, we comprehensively analyzed the expression profiles of TCGA-COAD cohort and Human Ageing Genomic Resources database, finally screened out 8 CS-related DEGs. Subsequently, we identified 237 CSRLs using the Pearson correlation method, 3 of which were prognostic CSRLs, named LINC02257, MYOSLID, and AC025165.1. LINC02257, as a enhancer RNA, has been demonstrated to be an independent prognostic factor for colon cancer patients (47). Moreover, LINC02257 was considered to be an independent prognostic biomarker for colorectal adenocarcinoma via the PI3K-Akt signaling pathway (48). A previous study reported MYOSLID was considered as an oncogene for gastric cancer (49). Besides, MYOSLID can be used to predict clinical outcomes in colon cancer patients (50). MYOSLID knockdown has been reported to lead to a decrease in CD4+ T cells in colorectal cancer cells, which may play a role in regulating immunity to colorectal cancer (37). However, to our knowledge, the correlation between AC025165.1 and colon cancer has not been reported.

We developed a novel prognostic 3-CSRLs model for colon cancer, which could provide an effective basis for clinicians to estimate the prognosis of colon cancer patients. Considering that senescent cells secrete a variety of proteins, such as inflammatory cytokines, chemokines, and growth factors, etc., which lead to an antitumor immune response through recruitment of immune cells (51, 52). Moreover, senescent cells can reshape surrounding tissue by regulating the properties of neighboring cells, including stromal and immune cells (53). Therefore, we also explored the immune microenvironment characteristics of CSRLs on colon cancer. Patients with low-risk had more immature immune cells such as naïve B cells or immunosuppressive cells such as regulatory T cells compared to those with high-risk. CD4+ T cells are known to play an important role in tumor immunity, which offer a promising strategy for immunotherapy of colon cancer (54). NK cells, as cytotoxic innate lymphocytes that eradicate tumor cells, induce a durable anti-tumor immune response, which is a priority in cancer immunotherapy (55). We observed a significant decrease in CD4+ T cells and NK cells in the high-risk group, and we speculated that the function of CD4+ T cells and NK cells may be relatively suppressed in the high-risk group. In addition, high-risk patients had a high level of M0 Macrophages that is associated with unfavorable survival, meaning CSRLs prognostic model can predict patient outcomes at the immune cell level. Because it is unidentified in clinical work to determine which colon cancer patients benefit from chemotherapy, this often leads to the misuse of chemotherapy drugs. In our study, the expression levels of multiple immune checkpoints in high-risk group were higher than those in low-risk group. Thus, it may be possible to improve outcomes in high-risk patients by enhancing their immune reactivity (56). Taken together, the CSRLs prognostic model reflected a different immunological microenvironment in colon cancer patients with diverse prognosis, and had a better predictive performance for immunotherapy.

Aging and diet are two of the most important risk factors for colon cancer and can enhance an oxidative state in the colon (57). Guo et al. found that senescent cells promote the formation of colon cancer by secreting GDF15 (35). Similar to other studies (37, 38), our study showed MYOSLID promoted the proliferation of colon cancer cells, and overexpression of MYOSLID prognosticated poor prognosis in colon cancer patients. MYOSLID was first reported to promote vascular smooth muscle differentiation (58). Many studies have reported MYOSLID as a prognostic factor for multiple cancers, such as head and neck squamous cell carcinoma (59), gastric cancer (49), and osteosarcoma (60). MYOSLID has been reported as a prognostic factor for colorectal cancer as a hypoxia-related lncRNA (38). However, what role MYOSLID plays in CS have not yet been reported. To the best of our knowledge, our study was the first to demonstrate that MYOSLID as a prognostic CSRL for colon cancer, and that knockdown of MYOSLID inhibited CS and growth of colon cancer. As mentioned earlier, there are two sides of CS that promote or antagonize the progression of colon cancer (34, 35). Moreover, there is a strong relationship between CS and TME. Chen et al. suggested that overexpression of INHBA is positively associated with poor prognosis in colorectal cancer, as well as regulating CS of colorectal cancer cells by mediating immune evasion in TME (61). Recent study found that NOX4, as a CS-related gene in colorectal cancer, may be a key factor in driving colorectal cancer resistance by altering TME (62). These findings give a hint that MYOSLID may promote tumor proliferation by mediating CS through regulation of immune microenvironment, but more evidence is still needed.

The present research has some shortcomings. Firstly, the original data for establishing the CSRLs prognostic model were only retrieved from the TCGA database. Additionally, other external datasets and external validation with clinical data are still needed to confirm the reliability and accuracy of the model. Moreover, the prognostic efficacy and underlying mechanisms of this model still require further study through real clinical data and basic experiments. Lastly, the mechanism of how CS regulates the development of colon cancer is still unclear and needs to be explained through additional studies.

## 5 Conclusion

Overall, we established a CSRLs prognostic model that could prognosticate the survival outcomes of colon cancer. The CSRLs prognostic model could effectually reflect the immune microenvironment characteristics and genomic mutation of colon cancer and the effect of immunotherapy and chemotherapy drugs. Finally, we discovered that MYOSLID could influence the biological function and CS of colon cancer. These suggested that CSRLs could be new biomarkers in diagnosis and prognosis of colon cancer.

## Data Availability

Publicly available datasets were analyzed in this study. This data can be found here: All data sets used in this study are publicly available on the UCSC Xena platform.

## Supplementary material

Supplementary Figure 1 Kaplan-Meier survival analyses of the relationship between the expressions of CSRLs with patients’ OS.

Supplementary Figure 2 Venn diagram.

Supplementary Table 1 The clinical information of the entire cohort.

Supplementary Table 2 The sequences of MYSOLID-ASOs.

Supplementary Table 3 The primer sequences used for q-PCR.

Supplementary Table 4 The information of 572 DEGs between normal and tumor tissues.

Supplementary Table 5 The relationship between CS-related DEGs and lncRNAs.
