# Supplementary figures and images for: Prognostic cellular senescence-related lncRNAs patterns to predict clinical outcome and immune response in colon cancer
Source: Front Immunol. 2024 Sep 2;15:1450135. doi: 10.3389/fimmu.2024.1450135 (PMC11443174; doi:10.3389/fimmu.2024.1450135)

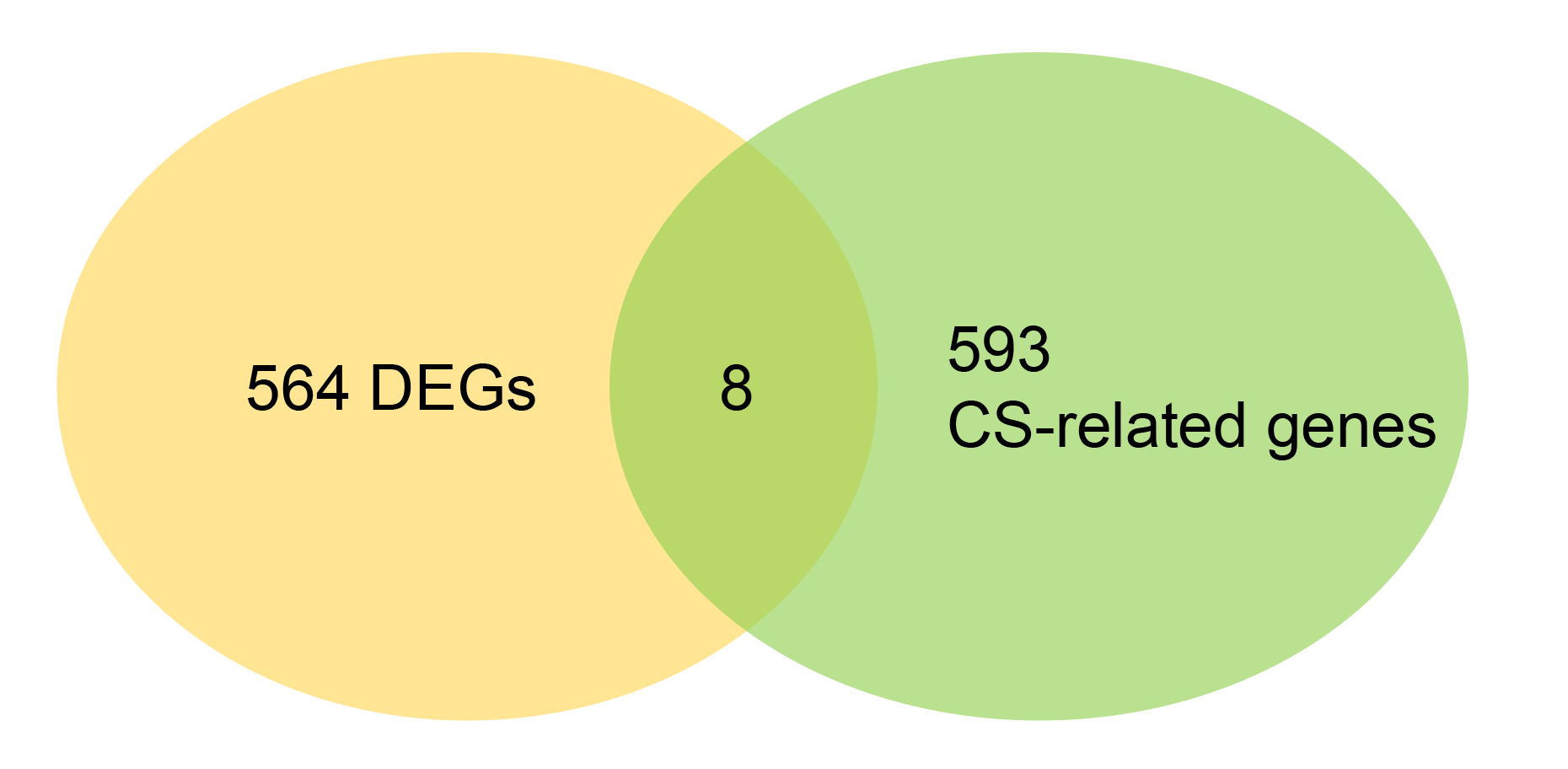

Supplement: Supplementary Figure 1 — Kaplan-Meier survival analyses of the relationship between the expressions of CSRLs with patients’ OS. [file Image1.tif]

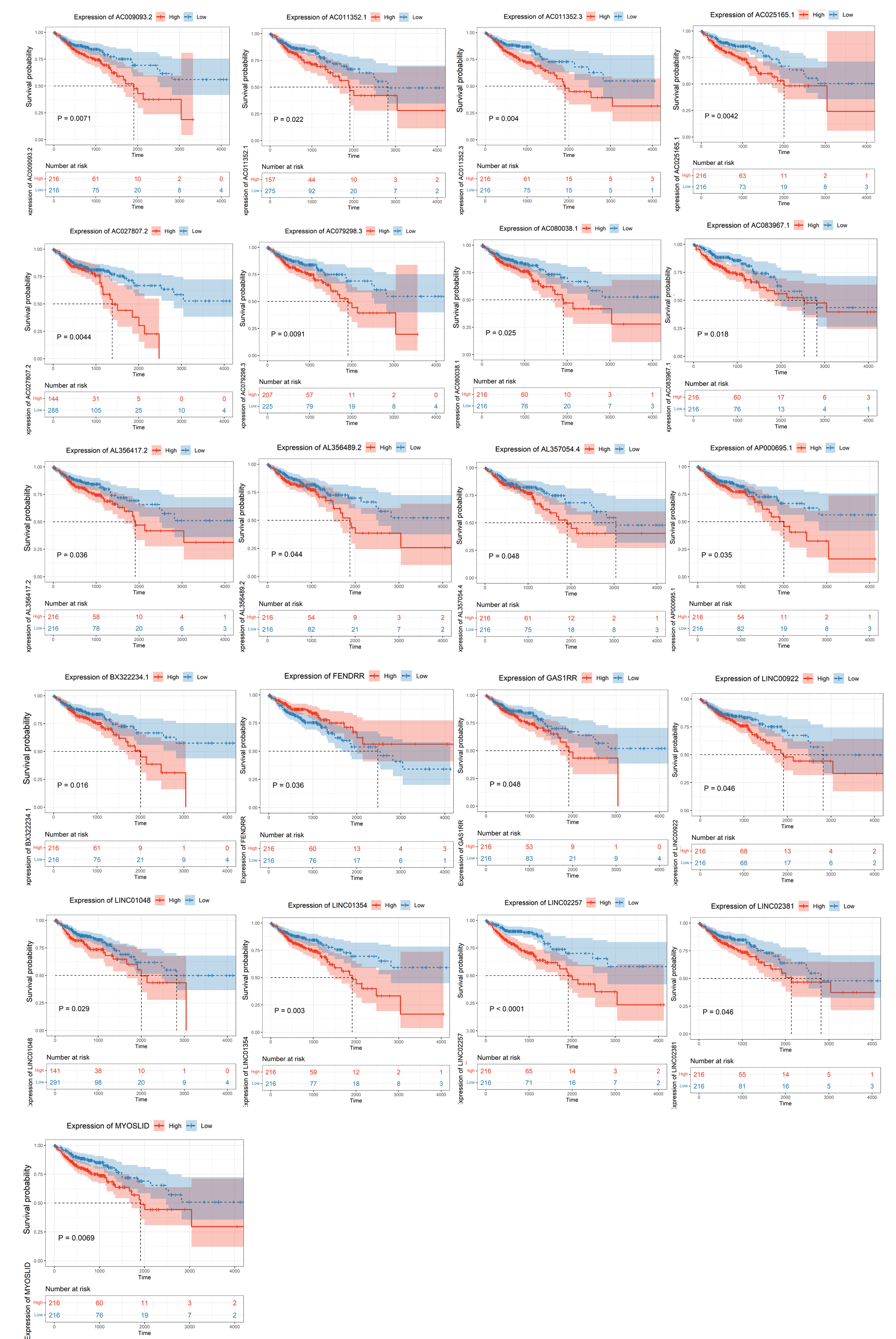

Supplement: Supplementary Figure 2 — Venn diagram. [file Image2.tif]
